# Society for Bettering the Condition of the Poor

**Published:** 1801-08

**Authors:** 


*The following, or ſimilar, Addreſſes are diſtributed in the Metropolis, and many other Parts of the Kingdom.*

---

### SOCIETY FOR BETTERING THE CONDITION OF THE POOR.

NOTWITHSTANDING the advantage of Inoculation for the Small-pox, it is a melancholy fact that the poor do ſtill ſuffer very greatly by that diſorder. The following addrefs from the phyficians, furgeons, and apothecaries of the medical hofpitals at Manchefter, on the fubject of Inoculation for the Cow-pox, may, it is hoped, have its effect in producing from *our* medical hofpitals, offers fimilar to that in the addrefs, and alfo in preparing the minds of the poor to take the full benefit thereof. Two families near Manchefter have lately inoculated for the Cow-pox many hundreds of the neighbouring poor, who have all recovered without any ficknefs to confine them a fingle day. Twenty of them were afterwards inoculated for the Small-pox; for a few days the ufual figns of infection were perceived on the arms, but foon difappeared, without communicating the Small-pox to any one of the twenty patients on whom this very fatisfactory experiment was made.

### ADDRESS TO THE POOR.

The experience of feveral years has fully proved, that inoculation for the Cow-pox is a certain *prefervative* againft the Small-pox; and is, befides, fo mild and fafe a diforder, when compared with the inoculated Small-pox, that it has been generally introduced among the better informed and more wealthy inhabitants, both of this kingdom and of various parts of Europe. In order, therefore, to imprefs ftrongly on the minds of the poor, the ufefulnefs and fuperior advantages of this new plan of inoculation, the medical gentlemen belonging to thefe charities have thought it their duty to ftate, in this public manner, the following obfervations, for the ferious perufal of all thofe poor perfons who feel *a proper affection for their offspring*, and who are defirous of promoting their own intereft and comfort.

1. *Inoculation* for the *Cow-pox* has been practifed for feveral years, with conftant fuccefs, in various parts of this kingdom.

2. It has never failed to prevent the infection of the natural Small-pox.

3. It may be communicated with fafety to perfons of every age and fex, and at all times and feafons of the year, with equal advantage.

4. The Cow-pox is much preferable to the Inoculated Small-pox, as being a milder and fafer difeafe, and not capable of infecting the perfons living in the fame family, or even fleeping in the fame bed.

5. It does not produce eruptions, which fcar and disfigure the face; and is feldom, if ever, attended with any other marks of the difeafe, than what appear on the arms from inoculation.

6. Neither fwellings, blindnefs, lamenefs, nor any other complaints, which are known frequently to be the confequences of the natural Small-pox, (and fometimes, though but feldom, of of the inoculated Small-pox) have been obferved to follow the Cow-pox.

7. Alarming fits frequently feize children when fickening of the Small-pox; and while cutting their teeth, this diforder often proves dangerous: But no fuch objections lie againft the Cow-pox.

8. So far from proving hurtful, delicate and fickly children are often improved in health by having paffed through this complaint.

9. Scarcely any remedies or attendance are required for the Cow-pox.

10. There is no neceffity for a courfe of phyfic either before or after inoculation.

11. The *time of the parents will not be taken up in attendance upon the sick*, to the injury of the fupport of the reft of the family: and to poor families, this is *an object of no small importance*.

The prejudices of the poor againft inoculation for the Small-pox, by which thoufands of lives have been annually faved, have been often lamented; but if they fuffer unjuft prejudices to prevent their laying hold of the advantages now offered to them by the inoculation of the Cow-pox, they will neglect the performance of a duty they owe to themfelves, to their families, and to fociety at large. For furely it is little lefs than criminal to expofe their helplefs children to the attack of fo terrible and fatal a malady as the Small-pox, when it may be readily avoided by the inoculation of fo mild, fimple, and fafe a difeafe as that of the Cow-pox.

N.B. All poor perfons, whofe affection for their families leads them to embrace this favourable opportunity, may have their children inoculated for the Cow-pox, at the hofpitals and difpenfaries, from twelve to one in the afternoon, every day in the week, (Sunday excepted) throughout the year. No time ought to be loft by the poor in freeing their families from the apprehenfion of the Small-pox, which daily increafes both in frequency and malignity throughout this town.

